# Impact factors and genetic characteristics of head lice infestation in schoolchildren: a cross-sectional study from 2018 to 2023 in central China

**DOI:** 10.1186/s13071-025-06825-9

**Published:** 2025-05-21

**Authors:** Yi-Tian Fu, Yuan-Ping Deng, Yue Xie, Hui-Mei Wang, Yan-Yan Peng, Guo-Hua Liu, Xiang Wu

**Affiliations:** 1 Department of Parasitology, Xiangya School of Basic Medical Sciences, Central South University, Changsha, 410013 Hunan China; 2 Research Center for Parasites and Vectors, College of Veterinary Medicine, Hunan Agricultural University, Changsha, 410128 Hunan China; 3 Department of Parasitology, College of Veterinary Medicine, Sichuan Agricultural University, Chengdu, 611130 Sichuan China

**Keywords:** Head lice, Prevalence, Impact factor, Schoolchildren, Duplex PCR

## Abstract

**Background:**

Head lice (*Pediculus humanus capitis*) infestation is a worldwide public health concern, especially in school-aged children. However, its main impact factors and genetic characteristics remain poorly understood in China. Hence, the purpose of the study was to explore the precise association between multiple impact factors and head lice infestation, characterize the genetic variation of the head lice, and develop a sensitive and specific mitochondrial (mt) DNA duplex polymerase chain reaction (PCR) for accurately distinguishing clades A and B.

**Methods:**

A cross-sectional study was conducted in Hunan Province, central China from January 2018 to July 2023. A total of 9254 schoolchildren from 48 primary schools in each administrative region were examined for head lice. Impact factors for infestation were analyzed using the data collected by a questionnaire. The mt *cytb* gene sequences of head lice collected in the current study were used for sequence analysis, then were added to the global pool to conduct the phylogenetic analyses. Primers designed on the basis of this gene sequence were used in duplex PCR to diagnose head lice clades A and B by amplicon size.

**Results:**

Head lice infestation was found in 93.8% (45/48) of the primary schools included in the study. Overall, 6.8% (630/9254) of the examined schoolchildren harbored head lice, with 94.6% (596/630) being girls. A total of 2132 adult head lice were collected from 630 infested cases. The impact factors for head lice infestation included gender, school location, family situation, per capita income, study mode, and hair washing per week (*p* < 0.01). However, season and age were not considered as impact factors for head lice prevalence (*p* > 0.05). Phylogenetic analysis based on mt *cytb* gene sequences showed that head lice are classified into two clades (A and B), with clade B being more dominant in Hunan Province, central China. The newly developed duplex PCR was able to differentiate clades A from B in China with 100% sensitivity and specificity.

**Conclusions:**

Our findings revealed that head lice infestation is mostly associated with poverty and poor hygiene in Hunan Province, central China. It is crucial to consider the simultaneous surveillance of head lice infestation in schoolchildren in regions with low level of socioeconomic status; however, datasets from other provinces are warranted to confirm the findings. It further showed that clades A and B are common in central China and that the latter has emerged and become the dominant one.

**Graphic Abstract:**

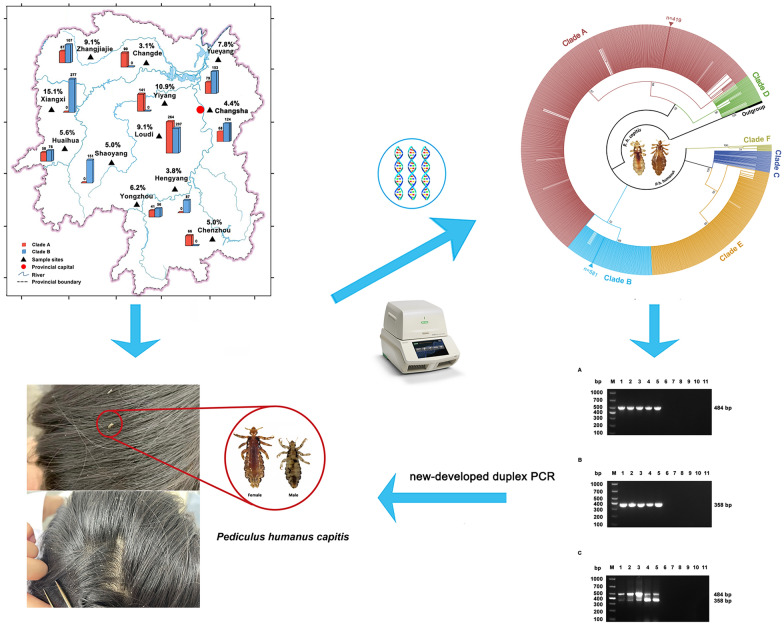

**Supplementary Information:**

The online version contains supplementary material available at 10.1186/s13071-025-06825-9.

## Background

Human pediculosis caused by human lice infestation is one of the most serious public health problems worldwide [1]. The head louse *Pediculus humanus capitis*, one of the most common human ectoparasites, inhabits human hairs and scalps and is transmitted via close contact or sharing hats and pillowcases [2]. Head lice infestations can cause sleeplessness, irritation, pruritus, and discomfort [3]. Besides, head lice have been proven to carry several pathogenic bacteria such as *Bartonella recurrentis*, *Bartonella quintana*, *Yersinia pestis* and *Acinetobacter* spp. [4–10]. A recent study corroborates the vectorial capacity of the head lice[10].

Head lice infestations are widespread throughout the world and affect individuals without discrimination of their socioeconomic status [11]. Head lice infestation affects hundreds of millions of people; in several areas, up to 78.6% of the populations were infected [2]. Schoolchildren are more prone to head lice infestation than the general populations around the world, with an average infection rate of about 25% (1977–2020) [12]. For examples, there is a prevalence of 44.3% in Cambodia [13] and of 54.1% in rural Thailand [14]. In southwestern Iran, the overall prevalence was 26.3%, with 25.1% and 36.9% in the urban and rural areas, respectively [15]. In a Mexican urban area, the overall prevalence was 28%, with 33.7% and 22.5% in girls and boys, respectively [16]. In the end of the 20th century in China, pilot surveys in Yunnan Province, Henan Province, Shandong Province, and Anhui Province showed 14.0%, 13.5%, 17.0%, and 19.2% of schoolchildren were infested in the rural areas, respectively [17–20]. These data collectively show that head lice infestation is a serious public issue of reemergence in schoolchildren worldwide.

Head lice and body lice are phylogenetically grouped into six distinct clades, A–F, on the basis of the mitochondrial (mt) *cytb* gene sequences, mt *cox1* gene sequences, or 12S ribosomal RNA (12S rRNA) gene sequences [21–24]. Interestingly, head lice appear in all clades, whereas body lice only belong to clades A and D [2]. Clade A of the head lice and body lice is cosmopolitan, whereas the rest are relatively restricted in geographical distribution [2, 21]. Clade B is mainly found in America, Australia, Israel, Algeria, South Africa, Saudi Arabia, Iran, and Western Europe [2, 24, 25]. Clade C is found in Africa (Ethiopia, Senegal, Gabon, and Democratic Republic of Congo) and Asia (Nepal, Pakistan, Malaysia, and Thailand) [2, 7, 26], while clade D is found in Ethiopia, Democratic Republic of Congo, and Zimbabwe [2]. Clade E is mainly identified in West Africa (Guinea, Mali, Senegal, and Nigeria) [2, 22], whereas the last-discovered Clade F is mainly from the head lice of Native American individuals in French Guiana, as well as from Argentina and Mexico [27]. Many studies have unequivocally shown that mt DNA sequences are invaluable genetic markers for the species identification and differentiation of ectoparasites, including lice [28–30].

Although pediculosis poses great threats to public health, there is a paucity on its data among Chinese schoolchildren [17–20]. Neither has the genetic diversity of head lice been investigated in China. It remains unclear what factors are associated with head lice infestation. Is head lice infestation associated with poverty and poor hygiene? In addition, although a multiplex real-time polymerase chain reaction (PCR) has been developed to identify and distinguish body lice from head lice [31], it is incapable of delineating different clades in detecting mixed infestations of clades A and B. There is an urgent need to develop reliable and sensitive molecular techniques such as duplex PCR for the specific differentiation for clades A and B of the head lice. Therefore, the objectives of this study were to (1) investigate the prevalence of head lice in schoolchildren in Hunan Province in central China, (2) estimate the impact factors of head lice infection in schoolchildren, and (3) identify the genetic characteristics and develop a sensitive and specific mt DNA duplex PCR for accurately distinguishing clades A and B of the head lice. Our findings have laid a solid foundation for the prevention and control of pediculosis in China.

## Methods

### Study design and setting

A large-scale cross-sectional study was conducted in the Hunan Province of central China from January 2018 to July 2023. The target schools were from each administrative city of Hunan Province. A total of 9254 schoolchildren, with 514 in Changde City, 501 in Changsha City, 925 in Chenzhou City, 1147 in Hengyang City, 792 in Huaihua City, 922 in Loudi City, 436 in Shaoyang City, 465 in Xiangxi Tujia and Miao Autonomous Prefecture, 735 in Yiyang City, 845 in Yongzhou City, 592 in Yueyang City, and 1380 in Zhangjiajie City were examined for head lice. We chose Hunan Province as the location of a full study because we carried out a pilot study that indicated the prevalence of pediculosis capitis in schoolchildren in Hunan Province was at least double that of the rest of the provinces of China. Hunan Province is situated between the northern latitudes of 24° to 30° and eastern longitudes of 108° to 114°. It has an area of approximately 210,000 square kilometers, with a population of 66 million. The number of children enrolled in primary schools at the time of the study was approximately 5.2 million. Permission to conduct the study was granted by the Education Office of Hunan Province. Furthermore, the study adhered to the guidelines outlined in the Strengthening the Reporting of Observational Studies in Epidemiology (STROBE) [32].

### Sample size

This study was a cross-sectional study, and the prevalence of head lice in schoolchildren was the primary indicator for estimating the sample size. The general formula* n* = *z*^2^ *p* (1–*p*)/*d*^2^ was used as indicated in the WHO health study manual [33] to determine the sample size, whereby *n* represented the sample size, *z* (1.96) is the standard deviation of the 95% confidence interval (95% CI), *p* is the estimated prevalence rate (50%), and *d* is the relative error permitted (3.5%) [34]. Since data on pediculosis prevalence in Hunan province were unavailable, a prevalence of 10% obtained in our pilot study was considered. The minimum sample size for each target school was 138, and the minimum sample size for this study was 6624 individuals. Ultimately, 48 elementary schools with a total enrollment of 9254 children (consisting of 4814 boys and 4440 girls) were included in the study. Schoolchildren from grades one to six with their guardians/parents’ consent were included.

### Questionnaires and samples collection

The participants at schools received informed consent forms about the head lice examination from the teacher addressed to the parents/caretakers, which also emphasized the importance of the prevention and treatment of head lice. The schoolchildren underwent head lice examinations after the informed consents were signed and returned. All children were willing to participate with their guardians/parents’ consent. Well-defined questionnaires were developed and used before the examination, covering a range of topics about participants including basic personal information (student number, age, and gender), personal hygiene (the frequency of hair washing per week), and socioeconomic status (family situation and per capita income). Questionnaires were completed preliminarily by each student about basic personal information (student number, age, and gender) with the help of his/her class teacher at school and then completed by parents or guardians after school about complex or sensitive items including personal hygiene (the frequency of hair washing per week) and socioeconomic status (family situation and per capita income).

**Fig. 1:**
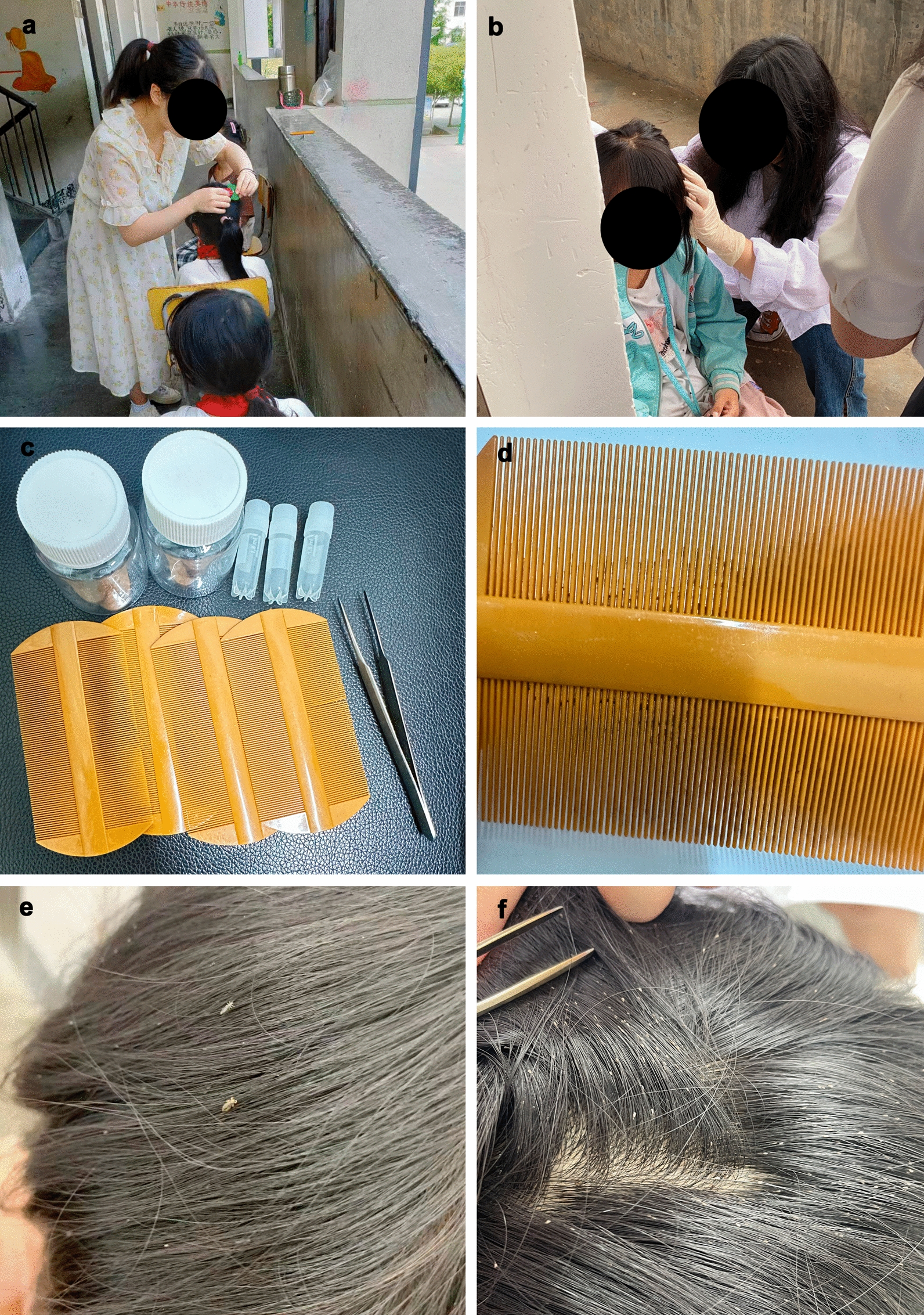
Head lice collection. **a**, **b** Visual inspection and combing method of *Pediculus humanus capitis* in schoolchildren. **c** Tools for head lice collection, including combs, tweezers, sterilized microcentrifuge tubes, and small plastic collection bottles. **d** Scalp, nit, or adult head louse residue in the used combs. **e** Adult head lice in the hair of a schoolchild. **f** Eggs of head lice in the hair of a schoolchild

The principal method for examining and identifying head lice was visually observing behind the children’s left and right ears, the scalp near the neck, or the hair 1 cm above the scalp and then combing hair by comb to separate nits, nymph, or adult of head lice from hair or scalp (Fig. 1). Head lice were collected by fingers or tweezers and immediately put in sterilized microcentrifuge tubes or small plastic collection bottles (Fig. 1). A child who had at least one living louse (nymph or adult) rather than only nits present on hair or scalp by visual examination was considered infested by head lice as previously described [2].

### Inclusion and exclusion criteria

The inclusion criteria required individuals to meet the following conditions simultaneously: studying in the same school for a long time, participating in normal activities of school, and being willing to participate in the investigation. The exclusion criteria include recent transfer students, unhealthy students who barely stay in school, students who did not agree to participate, or students whose parents or guardians did not agree to participate in the investigation.

### Statistical analysis

The factors analyzed in the current study were season, gender, age, school location, family situation, per capita income, study mode, and hair washing per week. In this study, a binary gender categorization (boys/girls) is adopted, which was designated at birth (“sex assigned at birth”). In addition, the per capita monthly income consists of “high” (≥ 10,000 RMB), “medium” (2000–10,000 RMB), and “low” (≤ 2000 RMB), demarcated on the basis of data from the Chinese National Bureau of Statistics. All statistical analyses were performed using SPSS 27.0 standard version for Windows (IBM Corporation). Chi-squared test was used to test the associations between head lice infestation and different risk factors. Univariate analysis was used to rate unadjusted association for each variable. Factors with a *p*-value < 0.20 in univariate analysis were included in the logistic regression model. Differences were considered statistically significant when the *p* was ≤ 0.05. Odds ratios (ORs) and 95% confidence intervals (CIs) were estimated to explore the strength of the association between head lice infestation and the risk factors.

### DNA extraction, PCR amplification, and DNA sequencing

Head lice were washed individually three times in physiological saline. Total genomic DNA was extracted from individual lice using the spin-column purification kit (Wizard^®^ SV Genomic DNA Purification System, Promega, Wisconsin, USA) per the manufacturer’s protocol, followed by proteinase K treatment. A total of 40 μl of elution buffer were used to elute the extracted DNA. The quality and quantity of the DNA were monitored by agarose gel electrophoresis and Qubit 2.0 Fluorometer (Thermo Scientific), respectively. PCR amplification and DNA sequencing were performed as previously described [35]. In brief, the partial mt *cytb* gene sequences (approximately 320 bp) of head lice was amplified by PCR using primers (CytbF1: GAGCGACTGTAATTACTAATC; CytbR1: CAACAAAATTATCCGGGTCC). PCR was performed in the C1000 Touch™ Thermal Cycler (BioRad, Hercules, CA, USA) in 25 μl including 2 μl DNA, 12.5 µl EmeraldAmp Max HS PCR Master Mix (Takara, 2X Premix), 0.5 μl each primer of 100 µM and 9.5 μl nuclease-free water. The thermal conditions included 95 °C for 15 min followed by 40 cycles of 95 °C for 1 min (denaturation), 56 °C for 30 s (annealing), 72 °C for 1 min (extension), and a final step at 72 °C for 5 min. A negative control of nuclease-free water was included in each PCR. PCR products were visualized by electrophoresis in 1% (w/v) agarose gel. Amplicons were sequenced in both directions (Sangon Company, Shanghai, China) using the same PCR primers.

### Sequence analysis and phylogenetic analysis

The newly obtained nucleotide sequences were compared with the nucleotide sequences deposited in the GenBank using the Basic Local Alignment Search Tool (BLAST: https://blast.ncbi.nlm.nih.gov/Blast.cgi). All sequences were aligned using MAFFT 7.429 [36]. Mutations including nucleotide substitution, deletion, and insertion were detected by chromatograms. Mt *cytb* gene sequences of head lice and body lice from different countries and geographic regions that deposited in GenBank combined with new representative sequences that had sequence variations delineated in the current study were included in phylogenetic analysis. A phylogenetic tree was built in neighbor-joining (NJ) with the Kimura 2-parameter model using MEGA 11 [37]. Bootstrap frequency (Bf) was calculated using 1000 bootstrap replicates with chimpanzee louse *Pediculus schaeffi* as an outgroup. Phylograms were drawn using the program FigTree v.1.4.

### Specific primers design and Duplex PCR

To design target-specific primers, the complete mt *cytb* gene sequences of different clades of head lice were sequenced using Illumina Hiseq 2500 at Majorbio (Shanghai, China). These complete mt *cytb* gene sequences were aligned using MAFFT 7.429 software [36] to obtain a consensus sequence, which was used to determine specific primers manually using the Primer Premier 5.0 (Premier Biosoft Interpairs, Palo Alto, CA). Specific primers are synthetized by Sangon Company (Shanghai, China). The PCR conditions were optimized for specificity by combinations of various annealing temperatures and magnesium concentrations. PCR reactions (in a volume of 25 μl) were performed in the C1000 TouchTM Thermal Cycler (BioRad, USA) and included 2 μl DNA, 12.5 µl EmeraldAmp Max HS PCR Master Mix (Takara, 2X Premix), 0.5 μl of each primer (two pairs), and 9.5 μl nuclease-free water. The thermal cycle was the same as described earlier; five other common sucking louse species (monkey louse *Pedicinus obtusus*, pubic louse *Pthirus pubis*, domestic pig louse *Haematopinus suis*, cattle louse *Linognathus vituli*, and rat louse *Hoplopleura kitti*) were included for confirmation of analytic specificity. In addition, negative (nuclease free water) controls were also included in each PCR run. The amplicons were sequenced as described above.

### PCR assay validation

In February 2022, 40 specimens of head lice were collected from schoolchildren in Loudi City, Hunan Province, China. Genomic DNA was extracted from whole individual head lice as described above. Of 40 samples amplified by duplex PCR, 20 representative positive samples were subsequently sequenced to validate the duplex PCR target specificity. Known positive (different clades of head lice and five other sucking louse species) and negative (no-template) control samples were also included in each PCR run.

## Results

### Prevalence of head lice in schoolchildren

**Fig. 2:**
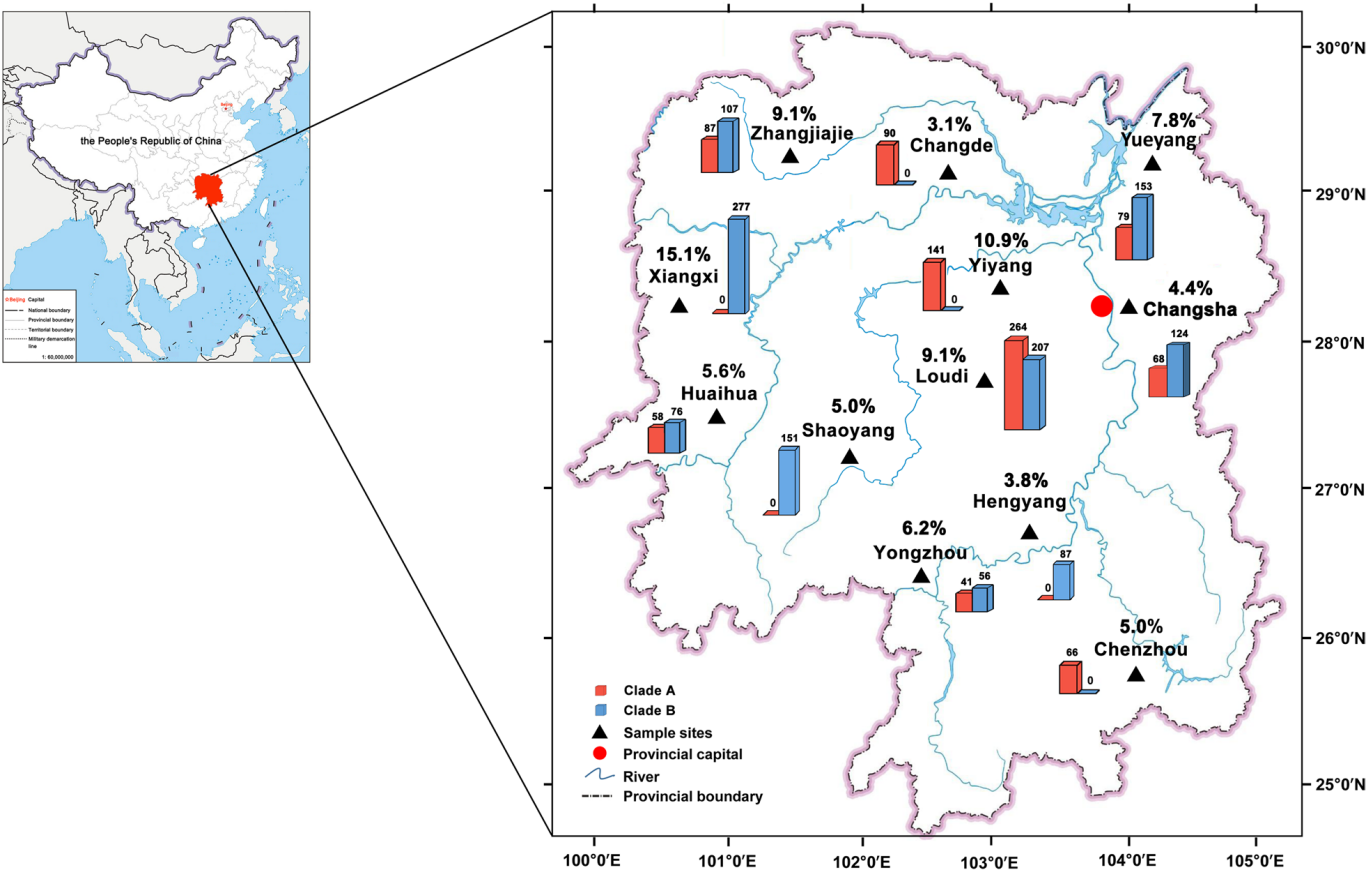
Prevalence and clade distribution of head lice in schoolchildren of each administrative city in Hunan Province, central China. The map of China in the top left corner is adapted from http://bzdt.ch.mnr.gov.cn/ [no. GS (2019) 1711], produced by the Ministry of Natural Resources of China

From January 2018 to July 2023, children in 45 out of 48 (93.8%) primary schools in Hunan Province, China, were found infested with head lice. Overall, head lice were detected in 630 of 9254 (6.8%; 95% CI 6.3–7.3%) of the schoolchildren (Table 1). The highest prevalence (15.1%) was in Xiangxi Tujia and Miao Autonomous Prefecture (Fig. 2), the region with the most economic backwardness in Hunan Province; followed by Yiyang City (10.9%), Zhangjiajie City (9.1%), and Loudi City (9.1%). The regions with the least prevalence were Changde City (3.1%), Hengyang City (3.8%), and Changsha City (4.4%); these three cities are the leading economic city in Hunan Province, whose per capita gross domestic product (GDP) stably ranks in the top four. In this regard, head lice infestation is relevant to economic development in Hunan Province. The head lice prevalence varied among four seasons, with the highest in winter (8.7%, OR = 1.5, *p* < 0.001), followed by summer (6.5%) and spring (6.4%), and the lowest in autumn (6.0%). Although head lice prevalence in children 6–8 years old (7.1%) was slightly higher than that in 9–11 years old (6.5%), these differences were not statistically significant. The head lice prevalence in girls (13.4%; 95% CI 12.4–14.4%) was significantly higher than that in boys (0.7%) (*p* < 0.001), with the largest OR of 21.8. The head lice prevalence in rural schools (11.7%; 95% CI 10.5–12.9%) was significantly higher than that in urban schools (4.7%) (*p* < 0.001) with an OR of 2.7. The head lice prevalence of the left-behind children (9.3%; 95% CI 8.2–10.4%) was significantly higher than the kids with parents at home (5.7%) (*p* < 0.001), with an OR of 1.7. There is a huge difference in GDP per capita among different families in Hunan Province; the prevalence ranged from 3.7% to 9.1% with statistical significance (*p* < 0.001). The head lice prevalence in school boarders (10.1%; 95% CI 9.1–11.0%) was significantly higher than nonboarding students (4.1%) (*p* < 0.001) with an OR 2.6. The head lice prevalence in hair washing once or twice per week (8.8%; 95% CI 8.0–9.6%) was significantly higher than hair washing thrice or more per week (4.7%) (*p* < 0.001) with an OR of 2.0.

**Table 1:** Prevalence and odds ratio (OR) of head lice infestation in schoolchildren in Hunan province, central China, 2018–2023

Factors	Variable	No. positive/no. tested	Prevalence % (95% CI)	*p*-Value	OR (95% CI)
Season	Spring	128/2013	6.4 (5.3–7.4)	0.656	1.1 (0.8–1.4)
	Summer	196/3025	6.5 (5.6–7.4)	0.492	1.1 (0.9–1.4)
	Autumn	136/2266	6.0 (5.0–7.0)		Reference
	Winter	170/1950	8.7 (7.5–10.0)	< 0.001	1.5 (1.2–1.9)
Age	6–8 years old	328/4637	7.1 (6.3–7.8)	0.322	1.1 (0.9–1.3)
	9–11 years old	302/4617	6.5 (5.8–7.3)		Reference
Gender	Boy	34/4814	0.7 (0.5–0.9)		Reference
	Girl	596/4440	13.4 (12.4–14.4)	< 0.001	21.8 (15.4–30.9)
School location	Rural	326/2792	11.7 (10.5–12.9)	< 0.001	2.7 (2.3–3.2)
	Urban	304/6462	4.7 (4.2–5.2)		Reference
Family situation	Left-behind children	241/2587	9.3 (8.2–10.4)	< 0.001	1.7 (1.4–2.0)
	Parents at home	389/6667	5.8 (5.3–6.4)		Reference
Per capita monthly income	High	102/2744	3.7 (3.0–4.4)		Reference
	Medium	168/2536	6.6 (5.7–7.6)	< 0.001	1.8 (1.4–2.4)
	Low	360/3944	9.1 (8.2–10.0)	< 0.001	2.6 (2.1–3.3)
Study mode	Day-student	211/5086	4.1 (3.6–4.7)		Reference
	School boarder	419/4168	10.1 (9.1–11.0)	< 0.001	2.6 (2.2–3.1)
Hair washing per week	Once or twice	420/4772	8.8 (8.0–9.6)	< 0.001	2.0 (1.7–2.3)
	Thrice or more	210/4482	4.7 (4.1–5.3)		Reference
	Total	630/9254	6.8 (6.3–7.3)

### Phylogenetic relationships and clade distribution of head lice

**Fig. 3:**
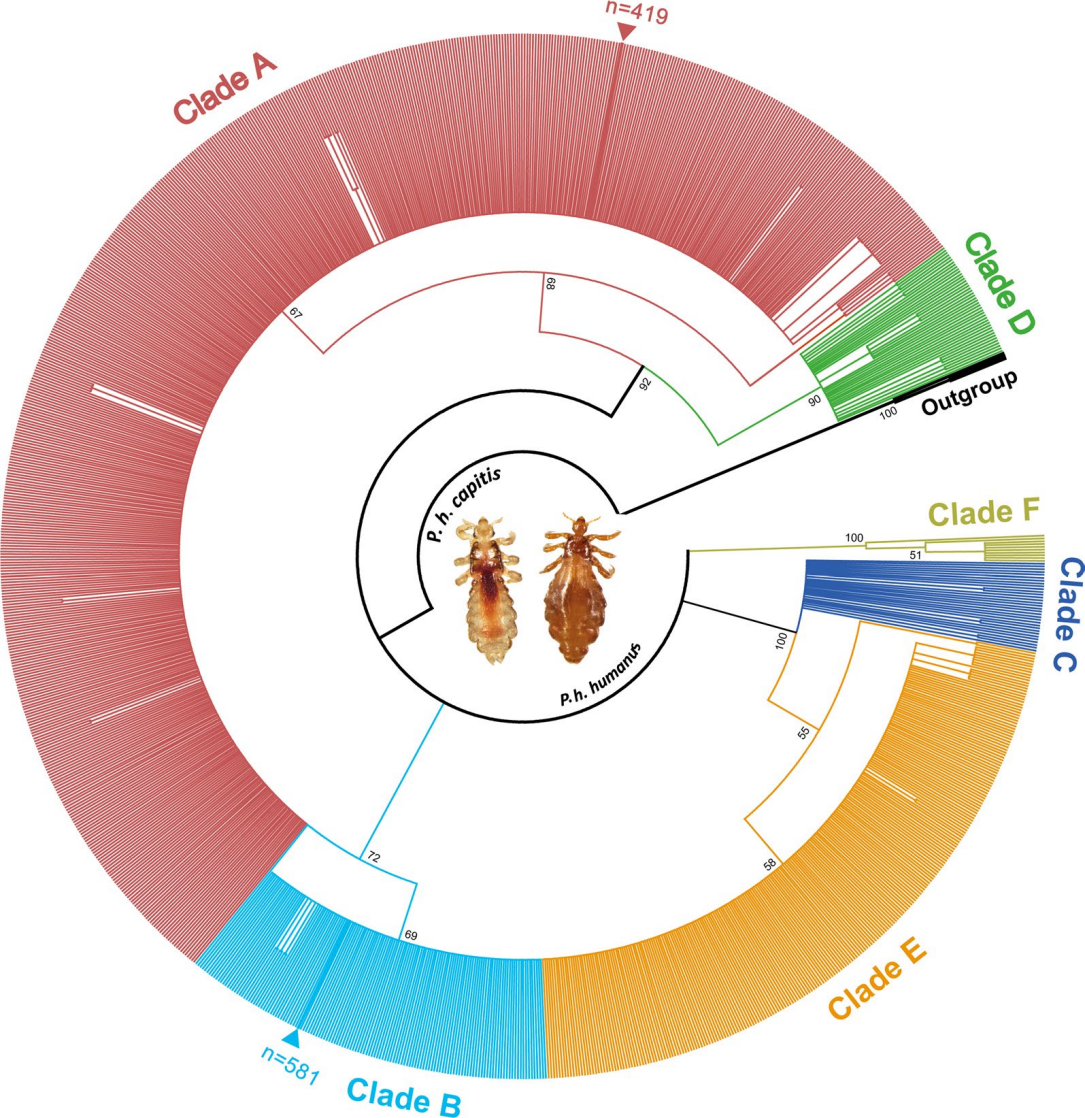
Phylogenetic tree of *Pediculus humanus* based on 1522 mitochondrial *cytb* gene sequences from different geographic countries and regions. The newly described sequences in the current study are indicated by *n* = 419 in clade A and *n* = 581 in clade B. Bootstrap frequency (Bf) was calculated using 1000 bootstrap replicates with *Pediculus schaeffi* as an outgroup

A total of 2132 adult lice were collected and sequenced in the current study. Sequence analysis results of mt *cytb* gene sequences showed there were only 1000 different sequences. The remaining 1132 sequences had no sequence variations and were identical with one of these 1000 sequences. The representative 1000 different sequences were identified as *P. h. capitis* by BLAST, with 41.9% and 58.1% being 98.7–100% and 98.3–99.8% identical to the sequences belonging to the clades A (GenBank no. KX444540) and B (GenBank no. AY316753), respectively. To further explore the phylogenetic relationship of *P. humanus* among different clades, the interclade phylogenetic tree was generated using 1522 mt *cytb* gene sequences comprising 1000 representative sequences delineated in the present study and 522 sequences of head and body lice from different countries and geographic regions available in the GenBank. It showed that *P. humanus* were divided into six divergent clades (Fig. 3). Our results also showed that all samples from the present study that represented head lice were placed into two distinct clades (A and B) (Fig. 3), further supporting the present of two clades in China. The number of head lice clades A and B in each administrative region is shown on Fig. 2. As we can see, the results indicated that both clades A and B are epidemic in Hunan Province. However, neither clades A nor B are prevalent in every administrative region in this province because clades A and B were individually identified in different geographic regions. Clade A was individually identified in Changde City, Yiyang City, and Chenzhou City, while clade B exclusively existed in Hengyang City, Shaoyang City, and Xiangxi Tujia and Miao Autonomous Prefecture (Fig. 2). The largest number of head lice (264 of clade A and 207 of clade B) was collected in Loudi City (9.1%, 84/922), which is not the region with the highest prevalence, indicating that each infested student (a total of 84) in the area was infested by at least five lice. Similarly, the smallest number of head lice (only 66 of clade A) was collected in Chenzhou City (5.0%, 46/925), which is not the region with the lowest prevalence. These phenomena revealed that the degree of head lice infestation of each schoolchild in each region is different.

### Specificity and sensitivity of the duplex PCR

We obtained the complete mt *cytb* gene sequences of head lice clade A (1074 bp, GenBank accession number: PV495020) and clade B (1071 bp, GenBank accession number: PV495021). The sequences variations were shown in Supplementary Table S1. Under the optimized conditions, primers HDAF/HDAR (Supplementary Table S2) amplified a product of 484 bp from clade A lice (Supplementary Fig. S1A), whereas primers HDBF/HDBR (Supplementary Table S2) amplified a product of 358 bp from clade B lice (Supplementary Fig. S1B). Further, a duplex PCR using primers HDAF/HDAR and HDBF/HDBR amplified two products of the expected sizes (484 bp and 358 bp) from mixed clades A and B lice (Supplementary Fig. S1C), which were confirmed by DNA sequences. The duplex PCR was specific to human head lice since it did not amplify the DNA of five other sucking lice and no-DNA controls (Supplementary Fig. S1). The limit of detection was 0.88 fg and 0.57 fg genomic DNA for clades A and B, respectively (data not shown). These findings unequivocally showed that the newly established duplex PCR was specific and sensitive, allowing for the detection and delineation of clades A and B of head lice in China.

The duplex PCR was then applied in testing 40 DNA samples. On agarose gels, each amplicon appeared as a single band of 484 bp or 358 bp in size (Supplementary Fig. S2). The 20 head lice samples had a PCR product of 484 bp in size, and they were identified as clade A lice. The remaining 20 head lice samples had a PCR product of 358 bp in size, and they were defined as clade B lice. No product was amplified from five other sucking lice samples and no-DNA controls (Supplementary Fig. S2). Among the 20 sequenced positive samples, 10 each belonged to the clades A and B, respectively.

## Discussion

Head lice represent the oldest human ectoparasites [38]. Despite the enormous progress in medicine and civilization, head lice infestation is still an important health issue worldwide [2, 21]. Currently, it was reported that head lice are more frequent in areas with low socioeconomic classes [26, 39–41]. In the present study, we found children in 93.8% (45/48) primary schools were infested with head lice in Hunan Province, central China. The reason why three schools are with no infestation may be the strict management system of these schools. The three schools are located in the Changsha (provincial capital of Hunan Province) urban area, and there are life teachers who regularly check the physical health and hygiene status of the students. The overall prevalence of head lice infestation in the present study was 6.8% (95% CI: 6.3–7.3%), significantly lower than the average prevalence (25%) of head lice infestation worldwide [12]. A global-scale evidence review generated from 436 papers found that head lice infestation in schoolchildren is influenced by temperature and climate, with the greater risk of infestation in the tropical regions of North and South America, Europe, Africa, and Asia [12]. This may be the reason why there is a relatively low total prevalence of head lice in Hunan Province.

Our results showed that the prevalence of head lice is not relevant with season and age but is affected by the gender, school location, family situation, per capita income, study mode, and frequency of hair washing per week. These results are mostly consistent with the results from an illustrative scale of evidence review based on 226 studies, which showed that hair washing frequency, father’s and mother’s education, and health instructor involvement are the factors with the greatest effects on infestation level, followed by the influence of family income and size; hairstyle factors, the existence of bathing facilities, bathing frequency, hair length, and education factors have weaker effects [42]. However, many studies showed that age was a significant factor [43–45], which was different from our results. This may be due to the potential for selection bias of participants as this study did not include kindergarten children.

In the present study, we analyzed eight different risk factors of head lice infestation. Girls have a significantly higher risk of head lice infestation than boys; this may be attributed to many factors such as longer hair length and closer interactions among girls [34, 46]. Schoolchildren in school from rural areas are more easily infested by head lice than those in urban locations because urban schools have more careful care for students, including good personal hygiene, suitable psychological care, and so on [47]. Usually, there are less teachers in rural schools than in urban schools in China, which results in the health and hygiene of some students being neglected. Similarly, most of the left-behind children are in rural areas without the company of parents; thus, they have a higher risk than those with parents at home because of the poor hygiene. Therefore, as school boarders, they are in communal life without special care from parents, leading to frequent contact with each other and neglected personal hygiene. Per capita monthly income is directly linked to socioeconomic status; people with high income usually have better quality of life, good education, and medical resources [48, 49]. This makes them able to avoid several diseases. Thus, schoolchildren whose parents with higher per capita monthly income have a lesser risk of infection. Moreover, hair washing influences the survival rates of head lice; thus, the prevalence of head lice is less when hair washing is more frequent [34]. Our results showed that personal hygiene behavior and family finances are key in determining the spread of head lice. In other words, poverty and poor hygiene may predispose children to head lice infestation. Low socioeconomic class is closely related to poverty and poor hygiene; the results in the present study indicate that socioeconomic status seems to be important in the spread of head lice, which is consistent with several studies [45, 49, 50], but contradicts the notion that head lice infestations do not favor any socioeconomic status [11, 51].

In the present study, we have confirmed that clade B (58.1%) has emerged as the predominant agent of head lice, which suggests that this ectoparasite potentially poses a new public health threat in Hunan Province of China where pediculosis is prevalent in schoolchildren. Clade B of head lice was initially confined to the New World, Europe, and Australia, and was later introduced to North and South Africa. It was previously considered of an American origin and brought to the Old World by Christopher Columbus’ exploration. However, increasing lines of evidence refute this hypothesis [24, 52]. Recently, clade B has also been detected in some Asian countries, including Israel and Saudi Arabia [24, 53, 54], and is emerging as a predominant lineage in the northwest of Iran [24]. Here, we have clearly showed that it has extended its geographic distribution to China.

Conventional PCR is limited in detecting mixed clades within a single child. To correct identification at the clade level, a multiplex real-time PCR has been developed to distinguish body lice from head lice [31]; it uses hypothetical protein (Phum_PHUM540560) rather than mt targets. Mitochondrial DNA marker is probably a better choice for a multiplex PCR owing to its stability and high copy number [55]. In the present study, we sequenced mt *cytb* gene sequences of clades A and B of head lice and successfully developed a duplex PCR for the identification and discrimination of clades A and B of head lice in China. Our results corroborate the notion that the mt *cytb* gene sequences are suitable DNA targets for distinguishing clade A lice from B lice [2, 21]. The newly developed duplex PCR was applied with 45 samples overall, comprising 20 each of clade A and clade B head lice and five other common sucking lice. The assay showed high specificity (100%) and sensitivity (100%) with a limit of detection of less than 1 fg genomic DNA among tested samples. The duplex PCR approach developed here does not require expensive equipment and reagents, and it is also simple and easy to use. Therefore, the duplex PCR developed in this study is applicable for clinical and epidemiological use, particularly for the confirmation and discrimination of clades A and B of the head lice. However, other clade lice samples such as clades C, D, E, and F had been not employed in this study because they have been not reported in China. Therefore, further validation is needed to see whether the assay is applicable to other geographic regions with the existence of other clades of head lice.

Head lice infestation remains an important health problem worldwide owing to multiplex factors including, but not limited to, cultural, social, economic, and public health issues [12]. It has significant psychological and medical impacts not only on the affected individuals themselves but also on their families and friends [56]. Therefore, parents of schoolchildren should regularly inspect them for and treat head lice infestation as needed. Better hygiene is the essential component for the control of pediculosis, such as washing hair with appropriate frequency; washing pillowcases and sheets regularly; keeping the head and body clean after outdoor activities; and avoiding sharing combs, hats, and scarves. In addition, health and education authorities are also encouraged to establish policies and strengthen publicity among teachers, parents, and schoolchildren to improve the understanding of pediculosis to reduce the incidence and prevalence of head lice infestation among schoolchildren.

Certain limitations of this study must be acknowledged. The sample size, sample set, and selection bias of participants may lead to an underestimation of prevalence. Further work is required to confirm that head lice infestation is associated with poverty and poor hygiene using larger numbers of specimens from broader geographical locations. In addition, this is a cross-sectional study with missing data (5.3%) due to loss of follow-up. In this study, missing data has been removed from our analysis. Therefore, in further analysis, missing data should be imputed using a multiple imputation method. Moreover, although we developed a new duplex PCR in the present study, other clade lice samples, such as clades C, D, E, and F, were not employed. Therefore, further validation is needed to determine whether the assay is applicable to other geographic regions with the existence of other clades of head lice.

## Conclusions

To our knowledge, this is the first study to investigate deeply head lice infestation in schoolchildren in China. Our results indicated that human pediculosis is still a common public health problem in schoolchildren in China and revealed that head lice infestation is mainly associated with poverty and poor hygiene. It is crucial to consider the simultaneous surveillance of head lice infestation in schoolchildren in regions with low socioeconomic status; however, datasets from other Chinese provinces are warranted to confirm the finding. It further showed that clades A and B are common in China and that the latter has emerged as the dominant clade.

## Supplementary Information

Supplementary file 1. Fig. S1 Analysis of duplex PCR amplification of mitochondrial *cytb* gene sequences using specific primers for (A: clade A; B: clade B; C: mixed DNA template of clades A and B) by agarose gel electrophoresis. Lane 1-5 represents genomic DNA of clade A, clade B and mixed DNA template of head lice, respectively. Lanes 6-10 represent genomic DNA from macaque louse *Pedicinus obtusus*, pubic louse *Pthirus pubis*, pig louse *Haematopinus suis*, cattle louse *Linognathus vituli* and rat louse *Hoplopleura kitti*, respectively. Lane 11 represents no-DNA control. M represents a DNA size marker (ordinate values in base pairs).

Supplementary file 2. Fig. S2 Analysis of PCR products amplified from representative louse samples using the newly-developed duplex PCR by agarose gel electrophoresis. Lanes 1-16 represent samples PH1, PH2, PH3, PH4, PH5, PH6, PH7, PH8, PH9, PH10, PH11, PH12, PH13, PH14, PH15, PH16, respectively. Lanes 17-21 represent genomic DNA from macaque louse *Pedicinus obtusus*, pubic louse *Pthirus pubis*, pig louse *Haematopinus suis*, cattle louse *Linognathus vituli* and rat louse *Hoplopleura kitti*, respectively. Lane 22 and 23 represent two no-DNA controls. Lanes 24 and 25 represent known clades A and B, respectively. M represents a DNA size marker (ordinate values in base pairs).

Supplementary file 3. Table S1. Nucleotide differences in the mitochondrial complete* cytb* gene sequences between clades A and B from Hunan province, China. Table S2. Species-specific primers used in this study.

## Data Availability

No datasets were generated or analyzed during the current study.

## Abbreviations

Mt: Mitochondrial
*Cytb*: Cytochrome b
*Cox1*: Cytochrome *c* oxidase subunit 1
12S rRNA: 12S ribosomal RNA
PCR: Polymerase chain reaction
95% CI: 95% confidence interval
Cm: Centimeter
Ml: Milliliter
Min: Minute
Sec: Second
BLAST: Basic local alignment search tool
NJ: Neighbor-joining
Bf: Bootstrap frequency
OR: Odds ratio
GDP: Gross domestic product
