# Supplementary figures and images for: Impact factors and genetic characteristics of head lice infestation in schoolchildren: a cross-sectional study from 2018 to 2023 in central China
Source: Parasit Vectors. 2025 May 21;18:184. doi: 10.1186/s13071-025-06825-9 (PMC12096793; doi:10.1186/s13071-025-06825-9)

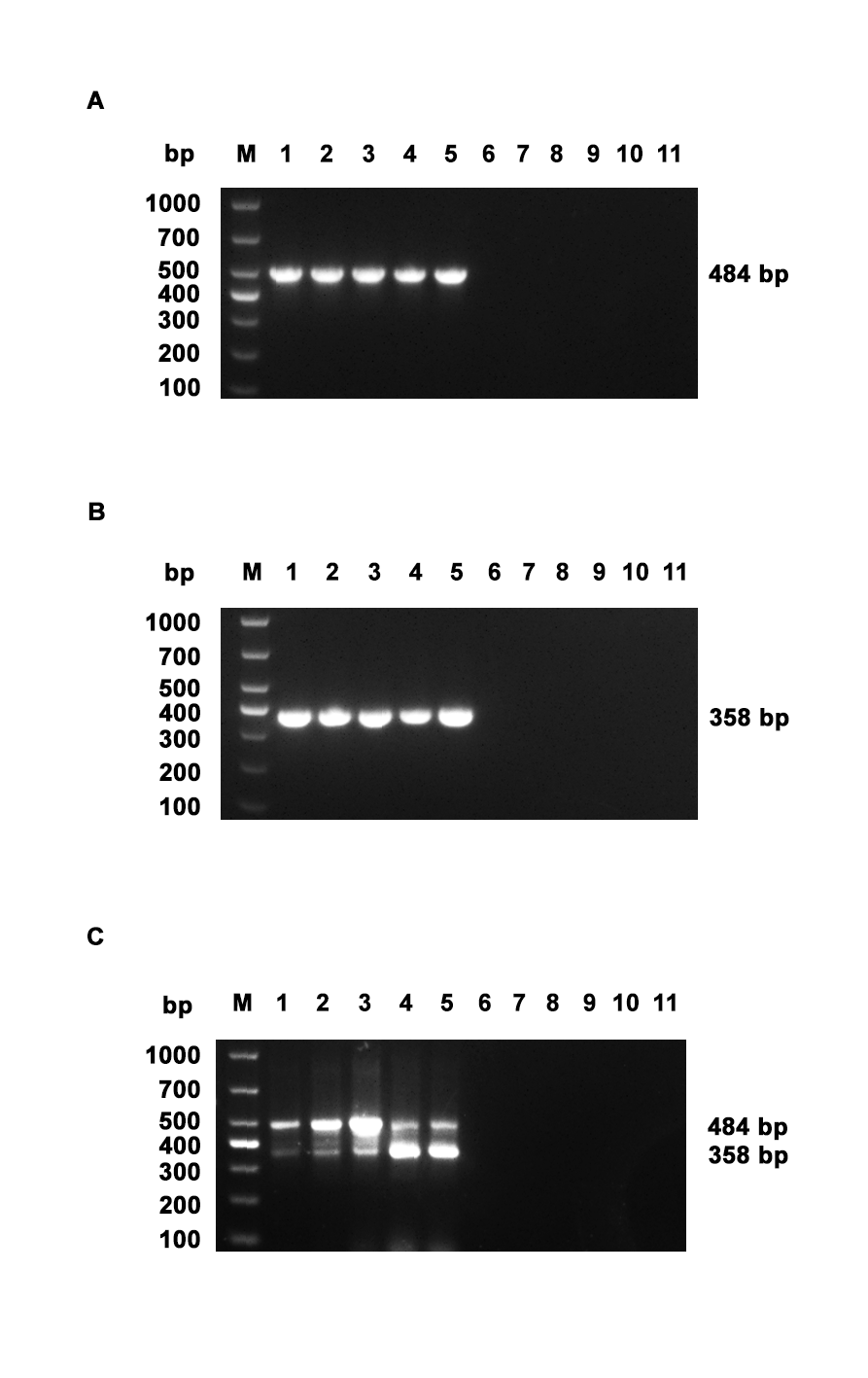

Supplement: Supplementary file 1 — Fig. S1 Analysis of duplex PCR amplification of mitochondrial cytb gene sequences using specific primers for (A: clade A; B: clade B; C: mixed DNA template of clades A and B) by agarose gel electrophoresis. Lane 1-5 represents genomic DNA of clade A, clade B and mixed DNA template of head lice, respectively. Lanes 6-10 represent genomic DNA from macaque louse Pedicinus obtusus, pubic louse Pthirus pubis, pig louse Haematopinus suis, cattle louse Linognathus vituli and rat louse Hoplopleura kitti, respectively. Lane 11 represents no-DNA control. M represents a DNA size marker (ordinate values in base pairs). [file 13071_2025_6825_MOESM1_ESM.tif]

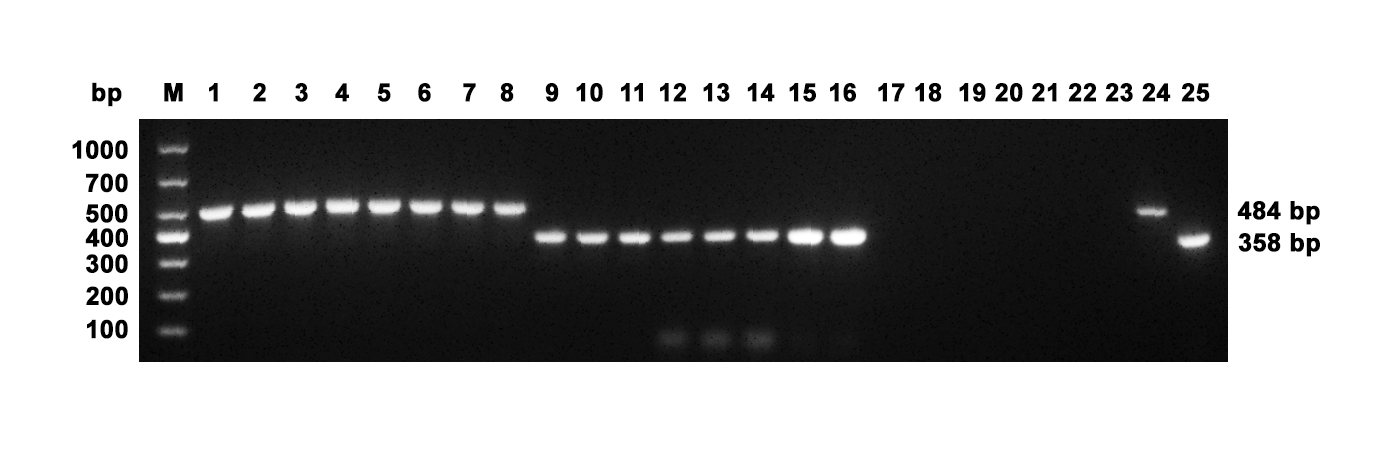

Supplement: Supplementary file 2 — Fig. S2 Analysis of PCR products amplified from representative louse samples using the newly-developed duplex PCR by agarose gel electrophoresis. Lanes 1-16 represent samples PH1, PH2, PH3, PH4, PH5, PH6, PH7, PH8, PH9, PH10, PH11, PH12, PH13, PH14, PH15, PH16, respectively. Lanes 17-21 represent genomic DNA from macaque louse Pedicinus obtusus, pubic louse Pthirus pubis, pig louse Haematopinus suis, cattle louse Linognathus vituli and rat louse Hoplopleura kitti, respectively. Lane 22 and 23 represent two no-DNA controls. Lanes 24 and 25 represent known clades A and B, respectively. M represents a DNA size marker (ordinate values in base pairs). [file 13071_2025_6825_MOESM2_ESM.tif]
