# Supplementary material for: Impact factors and genetic characteristics of head lice infestation in schoolchildren: a cross-sectional study from 2018 to 2023 in central China
Source: Parasit Vectors. 2025 May 21;18:184. doi: 10.1186/s13071-025-06825-9 (PMC12096793; doi:10.1186/s13071-025-06825-9)
Supplement: Supplementary file 3 — Table S1. Nucleotide differences in the mitochondrial complete cytb gene sequences between clades A and B from Hunan province, China. Table S2. Species-specific primers used in this study. [file 13071_2025_6825_MOESM3_ESM.docx]

**Table S1.** Nucleotide differences in the mt complete *cyt*b gene between clades A and B from Hunan province, China.

| Samples | Positions | | | | | | | | | | | | | | | | | | | | | | | | | | | | | | | | | | | | |
| --- | --- | --- | --- | --- | --- | --- | --- | --- | --- | --- | --- | --- | --- | --- | --- | --- | --- | --- | --- | --- | --- | --- | --- | --- | --- | --- | --- | --- | --- | --- | --- | --- | --- | --- | --- | --- | --- |
|  | 9 | 30 | 54 | 57 | 63 | 66 | 114 | 123 | 165 | 195 | 198 | 221 | 239 | 249 | 269 | 291 | 328 | 336 | 366 | 381 | 393 | 439 | 498 | 507 | 529 | 534 | 540 | 564 | 567 | 570 | 579 | 594 | 597 | 603 | 604 | 618 | 636 |
| clade A1 | A | T | A | C | A | T | T | G | T | G | G | C | C | C | T | A | C | A | T | A | A | A | G | T | G | A | G | C | C | C | T | A | A | T | T | A | C |
| clade A2 | A | T | A | C | A | T | T | G | T | G | G | C | C | C | T | A | C | A | T | A | A | A | G | T | G | A | G | C | C | C | T | A | A | T | T | A | C |
| clade A3 | A | T | A | C | A | T | T | G | T | G | G | C | C | C | T | A | C | A | T | A | A | A | G | T | G | A | G | C | C | C | T | A | A | T | T | A | C |
| clade A4 | A | T | A | C | A | T | T | G | T | G | G | C | C | C | T | A | C | A | G | A | A | A | G | T | G | A | G | C | C | C | T | A | A | T | T | A | C |
| clade A5 | A | T | A | C | A | T | T | G | T | G | G | C | A | C | T | A | C | A | T | A | A | A | G | T | G | A | G | C | C | C | T | A | A | T | T | A | C |
| clade A6 | A | T | A | C | A | T | T | G | T | G | G | C | C | C | T | A | C | A | T | A | A | A | G | T | G | A | G | C | C | C | T | A | A | T | T | A | C |
| clade A7 | A | T | A | C | A | T | T | G | T | G | G | C | C | C | T | A | C | A | T | A | A | A | G | T | G | A | G | C | C | C | T | A | A | T | T | A | C |
| clade A8 | A | T | A | C | A | T | T | G | T | G | G | C | C | C | T | A | C | A | T | A | A | A | G | T | G | A | G | C | C | C | T | A | A | T | T | A | C |
| clade A9 | A | T | A | C | A | T | T | G | T | G | G | C | C | C | T | A | C | A | T | A | A | A | G | T | G | A | G | C | C | C | T | A | A | T | T | A | C |
| clade A10 | A | T | A | C | A | T | T | G | T | G | G | C | C | C | T | A | C | A | T | A | A | A | G | T | G | A | G | C | C | C | T | A | A | T | T | A | C |
| clade B1 | G | C | G | T | G | C | C | A | A | A | A | A | T | T | C | G | T | G | A | G | G | G | A | C | A | G | A | T | T | T | C | G | G | G | C | G | T |
| clade B2 | G | C | G | T | G | C | C | A | A | A | A | A | T | T | C | G | T | G | A | G | G | G | A | C | A | G | A | T | T | T | C | G | G | G | C | G | T |
| clade B 3 | G | C | G | T | G | C | C | A | A | A | A | A | T | T | C | G | T | G | A | G | G | G | A | C | A | G | A | T | T | T | C | G | G | G | C | G | T |
| clade B 4 | G | C | G | T | G | C | C | A | A | A | A | A | T | T | C | G | T | G | A | G | G | G | A | C | A | G | A | T | T | T | C | G | G | G | C | G | T |
| clade B 5 | G | C | G | T | G | C | C | A | A | A | A | A | T | T | C | G | T | G | A | G | G | G | A | C | A | G | A | T | T | T | C | G | G | G | C | G | T |
| clade B 6 | G | C | G | T | G | C | C | A | A | A | A | A | T | T | C | G | T | G | A | G | G | G | A | C | A | G | A | T | T | T | C | G | G | G | C | G | T |
| clade B 7 | G | C | G | T | G | C | C | A | A | A | A | A | T | T | C | G | T | G | A | G | G | G | A | C | A | G | A | T | T | T | C | G | G | G | C | G | T |
| clade B 8 | G | C | G | T | G | C | C | A | A | A | A | A | T | T | C | G | T | G | A | G | G | G | A | C | A | G | A | T | T | T | C | G | G | G | C | G | T |
| clade B 9 | G | C | G | T | G | C | C | A | A | A | A | A | T | T | C | G | T | G | A | G | G | G | A | C | A | G | A | T | T | T | C | G | G | G | C | G | T |
| clade B10 | G | C | G | T | G | C | C | A | A | A | A | A | T | T | C | G | T | G | A | G | G | G | A | C | A | G | A | T | T | T | C | G | G | G | C | G | T |
| Samples | Positions | | | | | | | | | | | | | | | | | | | | | | | | | | | | | | | | | | | | |
|  | 662 | 675 | 691 | 693 | 717 | 720 | 750 | 759 | 771 | 774 | 798 | 819 | 825 | 828 | 849 | 852 | 891 | 896 | 913 | 916 | 918 | 924 | 926 | 936 | 945 | 960 | 969 | 972 | 975 | 1005 | 1006 | 1020 | 1024 | 1035 | 1050 | 1061 | |
| clade A1 | C | A | A | T | C | A | G | A | C | A | A | A | C | G | G | T | C | T | A | A | G | G | T | T | G | G | A | T | C | G | C | A | G | C | A | C | |
| clade A2 | C | A | A | T | C | A | G | A | C | A | A | A | C | G | G | T | C | T | A | A | G | G | T | T | G | G | A | T | C | G | C | A | G | C | A | C | |
| clade A3 | C | A | A | T | C | A | G | A | C | A | A | A | C | G | G | T | C | T | A | A | G | G | T | T | G | G | A | T | C | G | C | A | G | C | A | C | |
| clade A4 | C | A | A | T | C | A | G | A | C | A | A | A | C | G | G | T | C | T | A | A | G | G | T | T | G | G | A | T | C | G | C | A | G | C | A | C | |
| clade A5 | C | A | A | T | C | A | G | A | C | A | A | A | C | G | G | T | C | T | A | A | G | G | T | T | G | G | A | T | C | G | C | A | G | C | A | C | |
| clade A6 | C | A | A | T | C | A | G | A | C | A | A | A | C | G | G | T | C | T | A | A | G | G | T | T | G | G | A | T | C | G | C | A | G | C | A | C | |
| clade A7 | C | A | A | T | C | A | G | A | C | A | A | A | C | G | G | T | C | T | A | A | G | G | T | T | G | G | A | T | C | G | C | A | G | C | A | C | |
| clade A8 | C | A | A | T | C | A | G | A | C | A | A | A | C | G | G | T | C | T | A | A | G | G | T | T | G | G | A | T | C | G | C | A | G | C | A | C | |
| clade A9 | C | A | A | T | C | A | G | A | C | A | A | A | C | G | G | T | C | T | A | A | G | G | T | T | G | G | A | T | C | G | C | A | G | C | A | C | |
| clade A10 | C | A | A | T | C | A | G | A | C | A | A | A | C | G | G | T | C | T | A | A | G | G | T | T | G | G | A | T | C | G | C | A | G | C | A | C | |
| clade B1 | T | G | G | C | T | G | T | G | T | G | G | G | T | A | A | C | T | C | G | G | A | A | C | C | T | A | G | A | T | A | T | G | A | T | G | T | |
| clade B2 | T | G | G | C | T | G | T | G | T | G | G | G | T | A | A | C | T | C | G | G | A | A | C | C | T | A | G | A | T | A | T | G | A | T | G | T | |
| clade B 3 | T | G | G | C | T | G | T | G | T | G | G | G | T | A | A | C | T | C | G | G | A | A | C | C | T | A | G | A | T | A | T | G | A | T | G | T | |
| clade B 4 | T | G | G | C | T | G | T | G | T | G | G | G | T | A | A | C | T | C | G | G | A | A | C | C | T | A | G | A | T | A | T | G | A | T | G | T | |
| clade B 5 | T | G | G | C | T | G | T | G | T | G | G | G | T | A | A | C | T | C | G | G | A | A | C | C | T | A | G | A | T | A | T | G | A | T | G | T | |
| clade B 6 | T | G | G | C | T | G | T | G | T | G | G | G | T | A | A | C | T | C | G | G | A | A | C | C | T | A | G | A | T | A | T | G | A | T | G | T | |
| clade B 7 | T | G | G | C | T | G | T | G | T | G | G | G | T | A | A | C | T | C | G | G | A | A | C | C | T | A | G | A | T | A | T | G | A | T | G | T | |
| clade B 8 | T | G | G | C | T | G | T | G | T | G | G | G | T | A | A | C | T | C | G | G | A | A | C | C | T | A | G | A | T | A | T | G | A | T | G | T | |
| clade B 9 | T | G | G | C | T | G | T | G | T | G | G | G | T | A | A | C | T | C | G | G | A | A | C | C | T | A | G | A | T | A | T | G | A | T | G | T | |
| clade B10 | T | G | G | C | T | G | T | G | T | G | G | G | T | A | A | C | T | C | G | G | A | A | C | C | T | A | G | A | T | A | T | G | A | T | G | T | |

**Table S2.** Species-specific primers used in this study.

|  | Primer code | Primer sequence (5’-3’) | Product length (bp) |
| --- | --- | --- | --- |
| Clade A | HDAF: | TATAGCTCACATTATTCTCCTCCAC | 484 |
|  | HDAR: | AATTACTCTTACAACTTGACTTAGC |  |
| Clade B | HDBF: | TCTAGAAATCCTTTGGGGTTGGAGC | 358 |
|  | HDBF: | AGTAAGTTAAAGTTATTCGTCCTAC |  |
